# Clinical workflow for MR-only simulation and planning in prostate

**DOI:** 10.1186/s13014-017-0854-4

**Published:** 2017-07-17

**Authors:** Neelam Tyagi, Sandra Fontenla, Michael Zelefsky, Marcia Chong-Ton, Kyle Ostergren, Niral Shah, Lizette Warner, Mo Kadbi, Jim Mechalakos, Margie Hunt

**Affiliations:** 1 Department of Medical Physics, Memorial Sloan Kettering Cancer Center, 1275 York Avenue, New York, NY 10065 USA; 2 Department of Radiation Oncology, Memorial Sloan Kettering Cancer Center, 1275 York Avenue, New York, NY 10065 USA; 3 MIM Software Inc, Cleveland, OH 44122 USA; 4 Philips Healthcare, 595 Milner Road, Cleveland, OH 44143 USA

**Keywords:** Synthetic CT, MRCAT, Clinical workflow, Prostate cancer

## Abstract

**Purpose:**

To describe the details and experience of implementing a MR-only workflow in the clinic for simulation and planning of prostate cancer patients.

**Methods:**

Forty-eight prostate cancer patients from June 2016 - Dec 2016 receiving external beam radiotherapy were scheduled to undergo MR-only simulation. MR images were acquired for contouring (T2w axial, coronal, sagittal), synthetic-CT generation (3D FFE-based) and fiducial identification (3D bFFE-based). The total acquisition time was 25 min. Syn-CT was generated at the console using commercial software called MRCAT. As part of acceptance testing of the MRCAT package, external laser positioning system QA (< 2 mm) and geometric fidelity QA (< 2 mm within 50 cm LR and 30 cm AP) were performed and baseline values were set. Our current combined CT + MR simulation process was modified to accommodate a MRCAT-based MR-only simulation workflow. An automated step-by-step process using a MIM™ workflow was created for contouring on the MR images. Patient setup for treatment was achieved by matching the MRCAT DRRs with the orthogonal KV radiographs based on either fiducial ROIs or bones. 3-D CBCTs were acquired and compared with the MR/syn-CT to assess the rectum and bladder filling compared to simulation conditions.

**Results:**

Forty-two patients successfully underwent MR-only simulation and met all of our institutional dosimetric objectives that were developed based on a CT + MR-based workflow. The remaining six patients either had a hip prosthesis or their large body size fell outside of the geometric fidelity QA criteria and thus they were not candidates for MR-only simulation. A total time saving of ~15 min was achieved with MR-based simulation as compared to CT + MR-based simulation. An automated and organized MIM workflow made contouring on MR much easier, quicker and more accurate compared with combined CT + MR images because the temporal variations in normal structure was minimal. 2D and 3D treatment setup localization based on bones/fiducials using a MRCAT reference image was successfully achieved for all cases.

**Conclusions:**

MR-only simulation and planning with equivalent or superior target delineation, planning and treatment setup localization accuracy is feasible in a clinical setting. Future work will focus on implementing a robust 3D isotropic acquisition for contouring.

**Electronic supplementary material:**

The online version of this article (doi:10.1186/s13014-017-0854-4) contains supplementary material, which is available to authorized users.

## Background

Magnetic resonance imaging (MRI) is playing an increasingly important role in the management of patients undergoing radiotherapy for prostate cancer. It has been known for many years that the superior soft tissue contrast of MRI improves delineation of the prostate and adjacent normal tissues compared with CT. Imaging and segmentation of the prostate, using CT alone, overestimates the prostatic volume by 30-40% [1, 2] Furthermore, segmentation errors have been observed throughout the gland and especially at the apex, and base regions with CT-only segmentation [3–5]. Despite the superiority of MR for prostate delineation, MR has not been routinely or widely used for target definition in radiotherapy because of the challenges in accurately registering diagnostic MR images to the radiotherapy CT planning images. Recently, MR simulation platforms, including flat tabletops with indexing, external laser positioning systems (ELPS), and MR optimal immobilization, have been introduced and further enable the use of MRI as the primary or secondary imaging modality for radiotherapy planning.

Although MRI has been incorporated into the treatment planning process through registration with CT-acquired planning images (or CT + MR simulation), this approach has recognized limitations. The advantages of using MRI as the primary imaging modality include minimizing dosimetric errors introduced by mis-registration with the planning CT, or temporal changes in anatomy, such as bladder and rectum filling between the two scans, improving efficiency, reducing redundant imaging as well as reducing patient costs and inconveniences posed by the need for two scans. Although methods for performing MR as the primary imaging modality and planning (or MR-only workflow) have been developed, actual clinical implementation and workflows are still in their infancy with limited published studies [6–8]. To implement a MR-only workflow in the clinic, there are several requirements, including: a) synthetic CT images (syn-CT) generated from single or multiple MR image sets with high geometric and dosimetric accuracy, b) MR-only simulation and isocenter marking, c) MR images with sufficient soft tissue contrast for contouring both target and normal structures, and d) 2-D digitally reconstructed radiographs (DRRs) or 3-D reference images with sufficient bone, soft tissue, and/or implanted fiducial visualization to guide image-based patient setup and treatment.

In our recent publication, we retrospectively validated various steps required to perform MR-only simulation using a first commercial synthetic CT software called MRCAT (or MR for **C**alculating **At**tenuation) on a 3 T Philips Ingenia platform^1^ [9]. The validation steps included dosimetric validation between planning CT and MRCAT syn-CT, image-guidance validation between 2D-DRR and 3D-cone beam computed tomography (CBCT) and the MRCAT syn-CT, and planning image validation through evaluation of patient-induced susceptibility distortion in MRCAT syn-CT. In this study, the details and experience of implementing MR-only workflow in the clinic for simulation and planning for prostate cancer patients receiving external beam radiotherapy are described. These workflows are general enough that they can be adapted to other anatomical sites.

## Methods

**Fig. 1:**
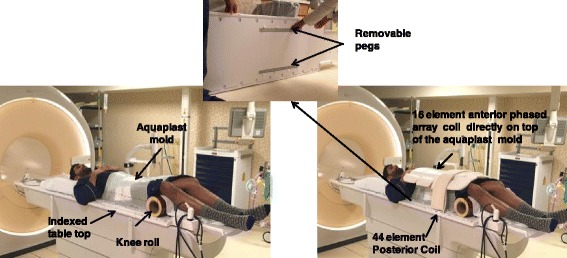
Prostate MR simulation setup (immobilization, knee roll and coil positioning) on a 3 T Philips Ingenia scanner. The inset shows the Philips flat table-top that was modified to match the CT pelvis board and include markings/ruler for reproducible setup as well as removable pegs for the Aquaplast mold

Prostate cancer patients (intact gland or post-operative prostate bed) undergoing external beam alone (8Gy × 5 or 1.80Gy × 40 fractions) or as a boost after a permanent brachytherapy implant (5 Gy × 5 fractions) were scheduled for MR-only simulation. Figure 1 shows the patient setup during MR simulation on the 3 T Ingenia Philips scanner. The MR setup matches our current CT simulation setup where a thermoplastic immobilization is placed anteriorly on the patient and indexed on a pelvis board. The inset of Fig. 1 shows the new oncology-specific flat tabletop from Philips that was modified to match the indexing on the CT pelvis board with markings for patient positioning and removable pegs to accommodate the MR compatible immobilization. The new tabletop replaces the original curved diagnostic table on the Ingenia scanner and also facilitates posterior coil placement to be located 1 cm closer to the patient during scanning, similar to what is achieved with the diagnostic table.

For patients with an intact prostate, three gold fiducial markers 3 mm length and 1.2 mm in diameter are routinely implanted into the prostate under ultrasound guidance prior to simulation. These markers were used to confirm and monitor the prostate position before and during high-dose radiation treatment using image guidance. In addition, as per our routine practice for hypofractionated stereotactic body radiation therapy (SBRT) treatments of intact prostate cases, a rectal hydrogel spacer was placed at the time of fiducial marker placement to achieve a separation between the prostate and anterior rectal wall to further minimize rectal toxicity in these patients [10, 11]. The spacer is best visualized using MRI compared with CT imaging and appears as a bright white signal in contrast to the surrounding anatomic structures. (See the green region of interest (ROI) in Fig. 3).

### MRCAT synthetic-CT

A detailed explanation of the MRCAT syn-CT algorithms has been provided in earlier publications and is summarized below [9, 12, 13]. MRCAT is the first commercial FDA-approved software for synthetic CT generation in the prostate. MRCAT CTs are generated from a single MR imageset called “MRCAT source,” a 3-D dual echo mDIXON FFE sequence based on a 2-point dixon reconstruction where three images are generated: water only (W), fat only (F) and an in-phase (IP) MR image. These 3 MR images are used in a classification algorithm to classify the image into soft tissue and bone class and are further divided into adipose, water, cortical bone and spongy bone. After classification into these different tissue types, every voxel is then assigned a bulk electron density. A dedicated exam-card is available for synthetic CT generation at the MR console where the imaging parameters are fixed and the user can only adjust the image stack position/location. The sequence is acquired in the transverse plane with 120 slices where the starting position of the stack is kept at the top of L4. Automatic failsafe steps are built into the MRCAT algorithm to detect problems with MRCAT classification and prevent MRCAT syn-CT generation. These failure detection modes are necessary for routine clinical use of the software. MRCAT syn-CT will not be generated for the following scenarios: (a) presence of hip prosthesis that affects the accurate classification of bone tissue, b) significant bone disease in the pelvis that compromises the accuracy of the bone classification, c) significant discrepancies from the bone model boundary conditions used in MRCAT post-processing that may arise from differences in patient positioning (such as with or without the use of a knee roll). In addition, if the patient size exceeds 50 cm in left-right (LR) or 30 cm in the anterior-posterior (AP) direction, MRCAT syn-CT may show a larger discrepancy. This limitation arises from the accuracy and acceptance of geometric distortion (< 2 mm) within this geometry.

In our previous publication we validated the accuracy for MRCAT syn-CT for MR-only planning in prostate [9]. Our analysis showed that the average dosimetric comparison between the original CT and syn-CT plans was within 0.5% for all structures [9]. The de-novo optimized plans on the syn-CT met institutional clinical objectives for target and normal structures. Patient-induced susceptibility distortion based on B0 maps was within 1 mm and 0.5 mm in the body and prostate, respectively, because of the very high readout or frequency bandwidth associated with the MRCAT source MR. DRR and CBCT localization based on MR-localized fiducials showed a mean standard deviation of <1 mm. End-to-end testing and MR simulation workflow was successfully validated [9].

### Simulation workflow

MR simulation begins with a therapist performing a daily morning quality assurance (QA) test with a Philips periodic image quality test (PIQT) phantom to monitor various parameters relevant to MR system performance and an ELPS phantom to verify the accuracy of patient translation from the laser isocenter to the MR isocenter. As part of acceptance testing of the MRCAT package, ELPS QA and geometric fidelity QA were performed and baseline values were set. A daily ELPS laser QA and biweekly geometric fidelity QA program has been setup at our institution along with the use of MRCAT syn-CT for clinical use.

**Fig. 2:**
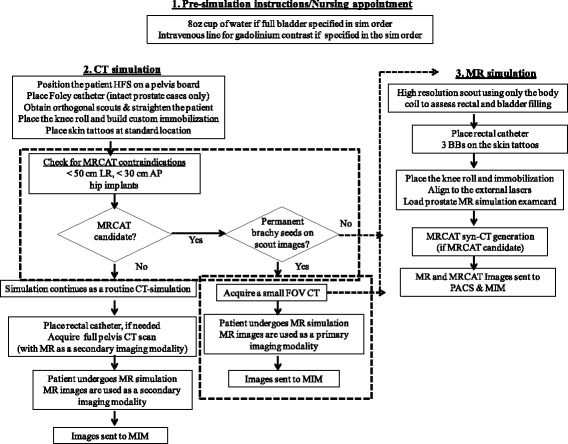
Flowchart explaining MR-only simulation workflow for the prostate. The dashed blocks represent the modification made to our existing CT + MR simulation workflow to accommodate a MR only workflow

MR-only simulation and planning for prostate initiates with a physician entering a simulation order for MR-only or MRCAT. A half-hour mold appointment is scheduled in the CT room and a half-hour simulation appointment is scheduled in the radiation oncology MR suite. Figure 2 shows the flowchart of our simulation processes. Our current combined CT + MR simulation process was modified to accommodate a MRCAT-based MR-only simulation workflow for the prostate. The dashed blocks in the flowchart represent the modification made to our existing CT + MR simulation workflow to accommodate a MR-only workflow.

The flowchart describes (a) pre-simulation (b) CT mold/simulation and (c) MR simulation process.

The patients are first taken to the CT room and steps for a routine CT-based simulation are followed to make the thermoplastic immobilization mold as shown in Fig. 1. A 15 cm diameter leg roll is placed under the knees, and the femur positions are adjusted to ensure that they are parallel to each other. Although not a strict requirement for MRCAT, the use of a knee roll was chosen to improve the MRCAT pass rate as the MRCAT bone model was generated with varying angulation of up to 15 cm in height. Once hardened, the mold is cut at three places to tattoo an initial reference point in the middle of the prostate. An orthogonal scout pair is acquired and MRCAT syn-CT feasibility, including patient size, or presence of hip implants is assessed. The orthogonal scouts are also used later to confirm the location of gold seed fiducials on MRCAT syn-CT DRRs. If the patient’s width in the left-right and anterior-posterior directions is greater than 50 cm and 30 cm, respectively, or if the patient has unilateral or bilateral hip implants, then the patient continues with the routine CT simulation. If the patient has had a permanent prostate brachytherapy implant, the therapists also acquire a small field of view (FOV) CT that will later help in distinguishing permanent seeds from the gold seed fiducials for performing MR-only treatment localization images.

In the MR simulator, using only the body coil, a quick low resolution (5x5x5 mm^3^) survey is acquired to assess patient straightening as well as bladder and rectal filling. Excess gas in the rectum is removed prior to simulation with the help of a rectal tube. The patient is positioned on the initial reference points marked in the CT by using the external laser positioning system in the MR simulator. The CT position is reproduced using the immobilization mold and an MR-compatible 15 cm diameter knee roll. Right before the coil is placed on the mold for MR scanning, three MR-compatible radio opaque Beekley™ markers (Beekley Inc., Bristol, CT 06010) (BBs) are placed on the tattoos so that they are visible on the large FOV MR images. These external markers are later used to create an isocenter at the triangulation point. Every effort is made to ensure that the BBs are aligned and are not moved when the coil and the Velcro belt are placed on top of the immobilization device. Please note that a coil bride is not used for our simulation because the immobilization mold is rigid enough to prevent modification of the patient outer. The patient is scanned with the MR sim exam card. A scanning guideline was created for the MR technicians and is shown in Table 1. The total acquisition time is approximately 25 min. Images are acquired in the following order to minimize potential motion discrepancies between the MR sequences: T2w sagittal, gold seed visualization, MRCAT source MR, T2w axial and T2w coronal. Additional T1w post-gadolinium contrast images are acquired if nodal volumes will be treated. While the MR images are acquired, an initial image quality assessment is done by the MR technologists to ensure good image quality for contouring and seed visualization for image guidance. MR technologists are instructed to repeat any acquisition during which significant motion was observed.

**Table 1:** MR simulation scanning guideline

Sequences	Coverage	Scan parameters
Sagittal T2 (For soft-tissue contouring)	Skin-to-skin (AP) Proximal bladder to rectum (SI) Middle of femoral heads (RL)	2D TSE FH x AP x RL =200x200x128 0.53 × 0.7 × 3 mm^3^ NSA = 3, TR/TE = (3000-6000)/70, BW = 250 Hz, FA = 90
Goldseed Axial (For fiducial identification)	Covering prostate and seminal vesicles	3D BFFE AP x RL x FH = 180x180x90 0.85 × 0.85 × 1 mm^3^ NSA = 2, TR/TE = shortest BW = 808 Hz, FA = 20
MRCAT Source MR Axial (For synthetic CT generation)	Skin-to-skin (AP) Skin-to-skin (RL) L4 below to proximal femur (SI)	3D FFE mDIXON AP x RL x FH = 324x324x120 1.7 × 1.7 × 2.5 mm^3^ TR/TE1/TE2 = 3.9/1.2/2.5, BW = 1072
Axial T2 small FOV (For soft tissue contouring)	Outer body (AP) Femoral heads (RL) L5 to anal canal (entire rectum) (SI)	2D TSE AP x RL x FH = 230x222x180 0.65 × 0.83 × 3 mm^3^ NSA = 3, TR/TE = (3000-6000)/100, BW = 246 Hz, FA = 90
Coronal T2 (For soft tissue contouring)	Middle of femoral heads (RL) Entire prostate, bladder neck, rectum (SI) Entire prostate, bladder neck, rectum (AP)	2D TSE FH x RL x AP = 180x180x128 0.59 × 0.78 × 3 mm^3^ NSA = 3, TR/TE = (3000-6000)/70, BW = 257, FA = 90

*Abbreviations*: *TSE* turbo spin echo, *NSA* number of signal averages, *BW* bandwidth, *FA* flip angle, *TR* relaxation time, *TE* echo time, *BFFE* balanced fast field echo, *FFE* fast field echo

### Contouring and planning workflow

The overall planning workflow includes contouring by the physician and planner in MIM Maestro™ followed by volumetric modulated arc therapy (VMAT) planning in Eclipse™. MR images used for contouring include MRCAT source MRs (W, IP), T2w small FOV axial, sagittal, coronal, MRCAT syn-CT, and gold seed fiducial sequence. Dose calculation involves the MRCAT syn-CT only. These images are automatically sent to MIM by the MR technologists. Because of the use of multiple MR images for contouring, an automated step-by-step process using a MIM workflow was created. The workflow begins with breaking the DICOM frame of reference (FOR) for the Goldseed and T2 axial MR image sequences and saving them as a new series for Eclipse export.. The remaining MRs not sent to Eclipse also require a break in the DICOM FOR to allow for independent adjustment of the registration The workflow continues with registration between all MR series to account for any intra-fraction motion that may have occurred during the 25-min simulation. This is done by first registering MRCAT source in-phase to all the remaining MR series (Goldseed, T2 axial, T2 sagittal, T2 coronal, and MRCAT source water) either based on implanted markers (for intact or post-implant cases) or bones (for prostate bed cases), and finally confirming the registration between MRCAT syn-CT and MRCAT source in-phase. At each step, the workflow pauses for the planner to evaluate the registration and adjust as needed (bony or fiducial-based). At any stage, the planner can adjust the registration manually or automatically using an ROI-assisted alignment invoked via a shortcut key.

Once all the fusions are completed, the workflow resumes, assigning MRCAT syn-CT as the primary image and loading the prostate structure template. At the end, the workflow creates various image page/visualization layouts for the physicians to aid in contouring using multiple MR series simultaneously. The final product with multiple page layouts, screen zoom, specific windlow/level and contour template is saved as a session for the physician to use in contouring. When the contouring is finalized and approved by the physician, the structure sets from the approved session are saved, and studies including the MIM registrations, are exported to Eclipse for planning.

For patients with implanted fiducials, an additional workflow is run by the planners to validate appropriate fiducial identification and segmentation by the MDs on the MRCAT syn-CT. This is done by performing a 2D-3D registration between the orthogonal CT scouts and the MRCAT syn-CT.

### Treatment localization workflow

Once the plan is finished and approved, 2-D DRRs for image-guided setup are generated from the MRCAT syn-CT using bony windows with fiducials displayed as ROIs. Please note that MRCAT syn-CT does not generate the physical fiducial marker. Rather, the fiducials are displayed as ROIs on the syn-CT as well as DRRs. On the treatment console, the patient is setup by matching the MRCAT CT DRRs with the orthogonal kV radiographs based on either fiducial ROIs for intact prostate cases or bones for prostate bed cases. A daily (hypofractionated cases) or weekly (standard fractionation) 3-D CBCT is also acquired and compared with the MRCAT syn-CT and MRCAT source MR, primarily to assess the rectum and bladder filling compared to simulation conditions. Because Varian on-board imager console can only display one primary image (MRCAT syn-CT), this step is done by the physicians in the Varian Eclipse™ Offline Review module, where they can change and display the primary image to the MRCAT MR for better soft tissue contrast.

## Results

The acceptance criteria for the daily laser QA on the MR simulator was < ± 2 mm, though the lasers agreed to within 1 mm tolerance. Geometric fidelity QA showed an accuracy or distortions <2 mm within ±20 cm geometry. MR scanner and the ELPS lasers were also calibrated to send the patient directly to the scanner isocenter based on the external lasers. A daily laser QA is performed by the RT therapist to check the tolerance for the ELPS lasers and also the distance between the external laser position and the bore isocenter. The DICOM nodes were also configured at the scanner to allow for a streamlined export of MRCAT syn-CT DICOM images from the MR console to the treatment planning system (TPS), including appropriate CT DICOM headers such as SOP UID, HU, rescale slope and modality. DICOM tags of the MRCAT images are also automatically set to indicate “CT” imaging modality to ensure that the TPS would accept them for dose calculation.

### MR-only simulation

A total of 48 prostate cancer patients treated between 06/2016 - 11/2016 were scheduled to undergo MRCAT. Out of these, 4 patients had hip prosthesis and 2 were found to exceed the MRCAT size limitations during their CT mold appointment. These patients subsequently underwent CT + MR-based simulation with MR as a secondary imaging modality. The remaining 42 patients represent the subject of this report and were successfully simulated, planned and treated with a MR-only workflow. Within this group, 25 patients were treated with SBRT to a prescription dose of 8 Gy × 5 fractions to the prostate, 8 were treated with SBRT of 5 Gy × 5 following permanent low dose rate interstitial implantation of Palladium-103 and 11 received salvage radiotherapy to the prostate bed in 1.80 Gy × 40 fractions. Two of the 42 patients had significant intra-fraction motion during the MR procedure that resulted in blurring of gold seed fiducials. These patients were still treated with a MR-only workflow. However, the first day of their treatment was used as a setup day, and the fiducial ROI was confirmed using the CBCT acquired on the setup day. Additional file 1: Figure S1 shows the comparison between gold seed sequences acquired in patients with and without significant motion artifact. As a result, an image quality assessment was implemented where the MR technologists review the MR images for motion and artifacts as they are acquired. The image quality assessment form is then loaded for each patient into ARIA as a post MR QA questionnaire document. The questionnaire consists of the following prompts to the therapists:

1. Were there any issues reconstructing the MRCAT syn-CT?
2. Are 3 external BBs clearly visible on MRCAT source MR? Are they also clearly visible on the same slice?
3. Are internal gold fiducials clearly visible on the fiducial sequence?
4. Is image quality on the small FOV T2 axial sufficient for identifying the prostate?
5. Was there patient movement during or between acquisitions?

The physicist is paged, and images are re-acquired if there is any concern. Additional file 1: Figure S2 shows the official questionnaire document in ARIA.

For patients with permanent brachytherapy seeds undergoing an external beam boost, a small FOV CT enables planners to distinguish between gold seeds and permanent brachy seeds. As mentioned earlier, the contouring and planning is still done with MRCAT syn-CT. The additional CT is only used as a secondary image to delineate gold seed fiducials for daily image guidance. Additional file 1: Figure S3 shows an example of MR and CT images with permanent brachytherapy seeds.

A total time saving of ~15 min was achieved with MR-based simulation as compared with CT + MR based simulation. While CT + MR simulation took a total of 1 h 15 min (45 min for CT simulation and 30 min for MR simulation), MR-only workflow could be achieved within 1 h (30 min for the mold appointment in CT and 30 min for MR simulation).

### MR-only contouring

**Fig. 3:**
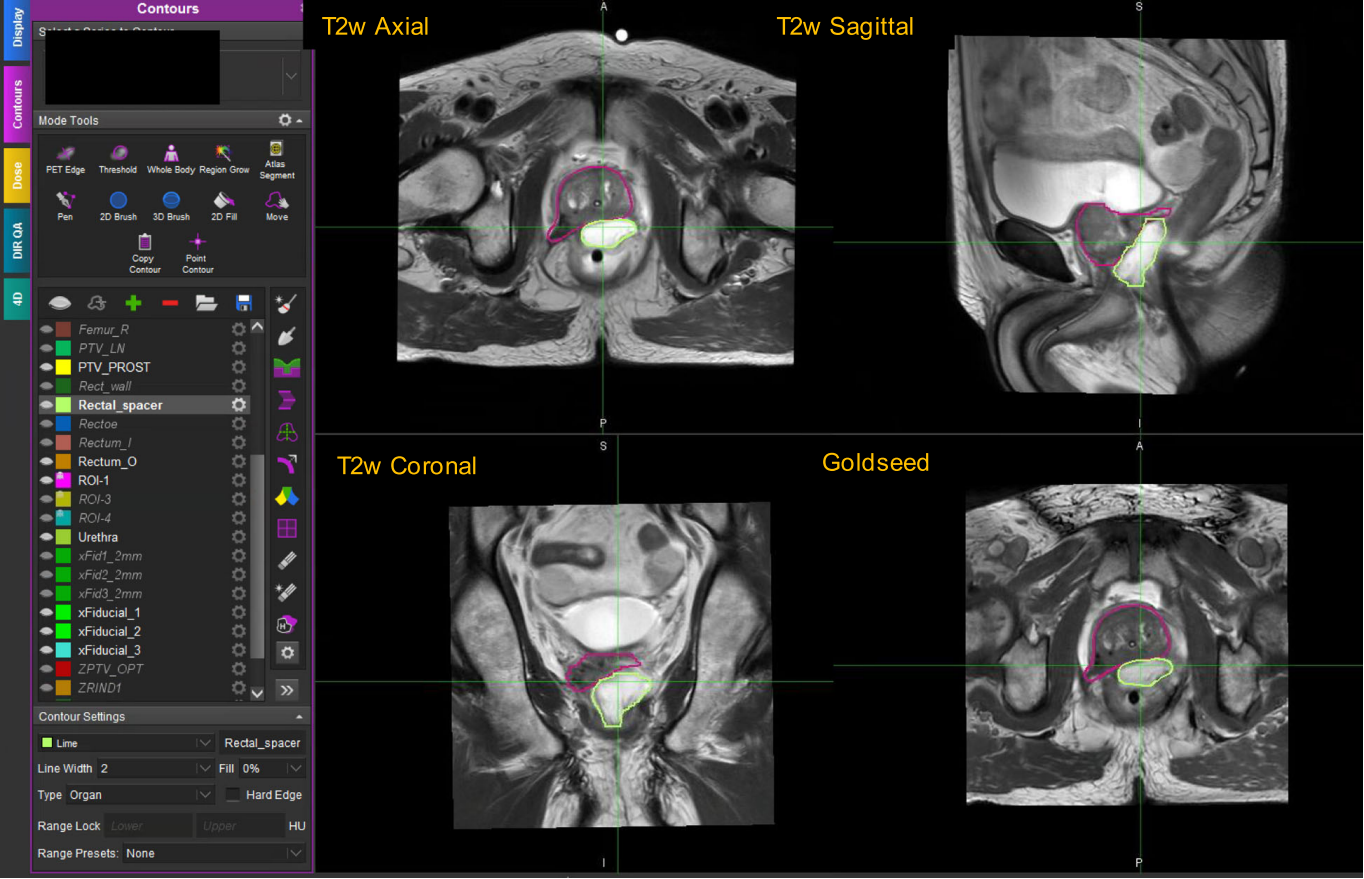
Contouring session in MIM displaying multiple MRs simultaneously. The display shows ROIs for PTV (pink) as well as rectal spacer (green)

The MIM workflow successfully streamlined the registration and contouring process. A single workflow was created to handle intact prostate gland, post-op prostate bed and post-implant brachytherapy cases. These three clinical scenarios have different requirements for image sequences. For example, the Goldseed sequence would not be acquired for prostate bed cases unless there was a residual disease with nearby landmarks such as surgical clips for fusion. For post-implant brachy cases, the workflow has been set up to easily handle the additional small FOV CT. Whenever nodal volumes are involved in any of the above cases, the workflow handles the post-contrast MR for nodal volume segmentation. Figure 3 shows an example of the image page layout created for the physician by the workflow to aid in contouring. Among different imaging layouts, a physics verification page was also created. The page displays T2w axial, Goldseed and MRCAT MR in-phase images. The purpose of this imaging layout is to assess that all the images are aligned with respect to the fiducials, and there is no intra-sequence misregistration. Additional file 1: Figure S4 shows an example of such a layout.

Physicians contour the CTV (prostate and seminal vesicles), bladder, bladder neck, bowel, urethra, rectum, and rectal spacer using native MR imaging protocol as shown in Fig. 3. The use of native MRs (T2 axial, sagittal and coronal) helps the physician to accurately identify the prostate base and apex for CTV contouring. The native acquisition also helps in identifying the bladder neck and rectal spacer along the three planes. If the FOV is not sufficient to contour a specific normal structure, such as bowel, the MDs make use of the large FOV MRCAT source image saved in a different image layout. Fiducials are identified on the Goldseed sequence and contoured using both the Goldseed and MRCAT source in-phase sequences. The workflow ensures that all contours are automatically saved on the MRCAT syn-CT even though physicians exclusively use only MR images for segmentation. Once the contouring is completed and approved by the physician, the planner opens the saved session and contours the remaining structures such as femur, bladder, rectum etc. The planner also identifies the most superior slice where the 3 BBs appear simultaneously and tags it as the plan isocenter slice. Additional file 1: Figure S5 shows an axial view of MRCAT syn-CT and MRCAT source MR used to identify the 3 external BBs.

**Fig. 4:**
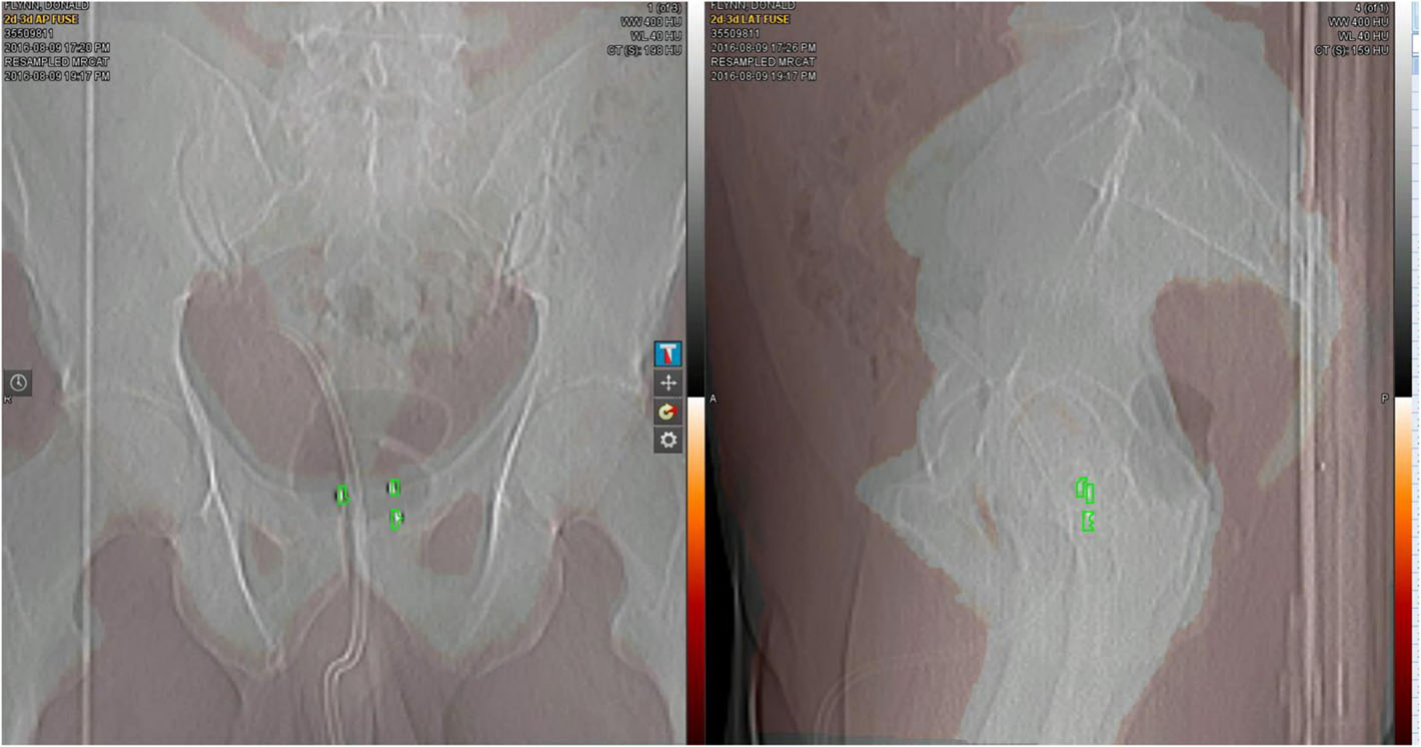
3D-2D registration between the MRCAT syn-CT and CT orthogonal scouts

Before saving the final structure set for Eclipse export, the planners confirm the fiducial contours by performing a 3D-2D registration between the MRCAT syn-CT and orthogonal scouts obtained during the CT mold appointment as shown in Fig. 4. The registration snapshot is saved into the patient document folder and subsequently evaluated by the plan checker during the initial plan QA check. Finally, the planner exports the MRCAT syn-CT, RT structure set, MRCAT source in-phase, Goldseed fiducial, T2 small FOV axial and their corresponding MIM registrations to Eclipse TPS. In Eclipse, the MRCAT source in-phase helps the planner and plan checker to confirm the isocenter position.

### MR-only planning

**Fig. 5:**
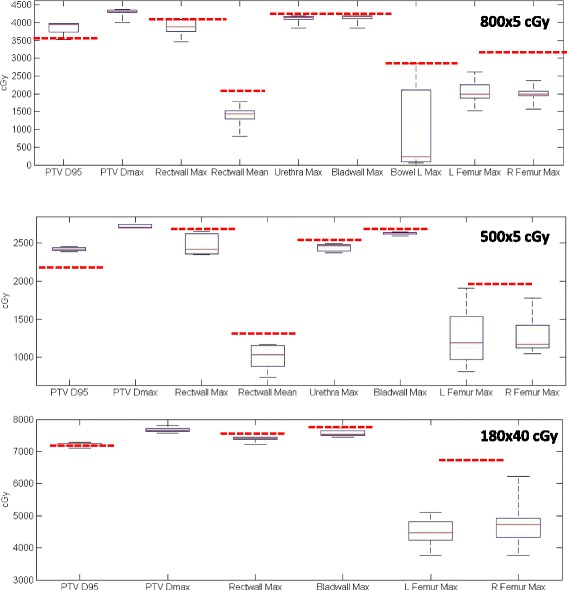
Dosimetric summary of all 42 cases planned with MR only workflow. Red horizontal lines represent our institution’s clinical objectives

A VMAT plan with two full 15 MV arcs was planned on MRCAT syn-CT using our institutional objectives that were created for CT-based plans. All 42 MRCAT cases were planned successfully and met the department’s clinical objectives (Fig. 5). The summary shows box plot evaluations of all relevant structures. The red horizontal dotted lines represent our institution’s clinical objectives developed for CT based plans but also applied to MRCAT cases.

### MR-only treatment localization

**Fig. 6:**
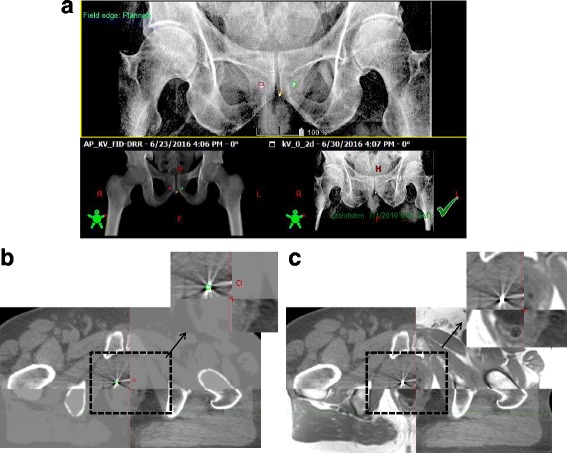
Treatment localization in Offline Review performed using: **a** 2-D kV DRRs from OBI and MRCAT syn-CT (**b**) 3-D match between CBCT and MRCAT syn-CT, (**c**) 3-D match between CBCT and MRCAT source MR using the MRCAT in-phase MR as a reference image

All MRCAT patients underwent successful image-guidance based on daily 2D bony DRR match for prostate bed cases or 2D fiducial match followed by 3D CBCT and intra-fraction monitoring for intact prostate cases. Figure 6a and b shows 2-D DRR and 3-D CBCT matching between MRCAT DRRs and kV radiographs and MRCAT syn-CT and CBCTs. Figure 6c shows CBCT matching when MR (MRCAT source MR) is loaded as a reference image in Offline Review.

## Discussion

In this study we have described the clinical workflows developed and implemented to perform MR-only simulation, planning and treatment localization for the prostate. Our experience based on the first 48 patients treated with MR-only workflow shows that MR-only planning is clinically feasible and can achieve similar, if not better, geometric and dosimetric accuracy as CT alone or CT + MR-based planning. Multiple checks and QA processes were implemented at various stages to streamline our clinical processes using MR images alone. Diagnostic quality MR images were obtained in the treatment position, which resulted in more precise target and normal structure contouring on MR as compared to CT. Contouring on MR was easier, quicker and more accurate compared with combined CT + MR images because there were no temporal variations in normal structures, e.g., bladder or rectum, that could potentially change the position of target such as seminal vesicles. Finally, the ability to load MR images as the primary reference image for CBCT localization enabled us to accurately position the patient during treatment delivery and implement a MR-only workflow that encompassed all steps from simulation to delivery. The MR-only workflow was successful for 42 out of 48 cases. The presence of hip implants and large body size prevented six patients from undergoing MR-only planning. Out of the the 42 patients who underwent MR-only planning and delivery, all met our institutional dosimetric objectives and completed treatment successfully.

Our current CT + MR simulation process was modified to accommodate a MR-only workflow that offered numerous advantages without disrupting the routine clinical workflow or increasing the simulation time. One of the major modifications to our existing clinical workflow for prostate radiotherapy was the inclusion of a knee roll for patient positioning to improve the MRCAT success rate. This modification was extended to our existing CT alone and the CT + MR workflow to provide consistency for the therapists. A part of our MR-only workflow still utilizes the CT room for making the patient-specific immobilization mold and determining candidate eligibility for the MR-only workflow based on initial orthogonal scouts. This step allows us to identify patients who will be ineligible for MR-only planning due to the current MRCAT limitations related to patient size and hip prostheses and easily and immediately transition to a CT-based simulation and workflow for them. In addition to determining candidates for the MR-only workflow, the orthogonal scouts provide information to verify the fiducial positions obtained from MR images during the planning stage, as shown in Fig. 4. The use of the CT room in the future can be avoided if the information regarding patient’s size and the presence of hip prosthesis is available and clearly documented before simulation. Please note that in this scenario it will be helpful to use fiducials that show a positive signal on the MR images.

Due to the large number of MR datasets used for contouring, there was a strong need for an organized workflow to streamline inter-sequence registration as well as generate automatic image layouts for physicians. The total time for MR simulation is 25 min, during which movement of the prostate and slight changes to bladder and rectal filling can occur. Our MIM contouring workflow allowed us to automatically break the DICOM FORs between the MR series and perform initial inter-sequence registration before contouring. The workflow also automatically saved the registration DICOM objects that could then be imported into Eclipse for later QA assessment by plan checkers. Our MR-only workflows provide a significant advantage for contouring both target and normal tissue structures from a single imaging modality through the creation of multi-image page layouts. Our dosimetric summary shown in Fig. 6 indicates that the plans produced using the MR-only workflow are comparable to the CT-based plans and that the MR-only planning has not compromised the quality of the plans in any way.

In our MR-only workflow we make use of relative isocenter positioning rather than absolute positioning through the use of initial reference tattoos placed during the CT simulation appointment. Placing MR compatible BBs on these tattoos allows recreation and identification of the isocenter on the MR images. The MRI platform currently does not provide the capability to perform absolute isocenter positioning for MR simulation, but third part software like MIM, or Eclipse, could be utilizedfor this purpose. To further streamline MR-only simulation and reduce simulation time, a waterbath in the vicinity of the MR scanner and a MR-compatible method for marking skin tattoos is needed. During the initial implementation stage we observed that the thermoplastic immobilization mold would dry slightly when taken from the waterbath to MR scanner. The use of a slow dry mold will certainly mitigate this issue. It is important to note that allowing mold to dry completely before imaging is necessary from a MRI safety perspective. A total timesaving of ~15 min was achieved with MR based simulation as compared to CT + MR based simulation. In the future, an additional timesaving of 15 min can be further achieved once mold making and patient tattooing is facilitated in the MR-sim suite itself.

Accurate delineation of fiducials is very important for fiducial-based image-guided treatment. Although a 3D b-FFE Goldseed sequence such as the one we use, generates sufficient contrast for fiducial identification, the sequence is very sensitive to patient motion. Movement during the acquisition can generate artifacts, which make it difficult to distinguish the gold fiducials from calcifications, or other image artifacts. As described in the results, two out of the 42 cases had significant blurring that made it challenging to delineate fiducials. (Additional file 1: Figure S1). Currently, this is one of the major limitations of a MR-only workflow. The development of motion-robust sequences may overcome this challenge in the near future. Until then, our strategy is to carefully evaluate and re-acquire the sequence when needed during simulation, or to use the first day of treatment as a setup day only to confirm the fiducial positions. Another challenge is to differentiate brachytherapy seeds from gold seed fiducials for post-implant brachy cases requiring external beam boost. For these cases, we are also acquiring a small FOV CT scan during the mold appointment. The MIM workflow was modified to include a registration between CT and Goldseed/MRCAT MR and to identify the fiducials as shown in Additional file 1: Figure S3. The gold seeds and permanent brachy seeds (Pd-103) are similar in size (3 mm length). Although the susceptibility is slightly different due to the difference in materials (gold vs. platinum/tungsten), the small size of the fiducials does not create sufficient difference between the two to allow differentiation. Until more robust methods for fiducial visualization and differentiation are obtained, we are acquiring an independent set of radiographic images during the CT mold appointment for each patient to ensure proper fiducial identification on the MR and accurate setup on the treatment machine. We are also investigating markers that show positive signal on both MR and CT/CBCT.

Our current MR-only workflow can be easily adapted to other anatomical sites that utilize fiducial (such as rectum, gynecological [gyn]) or bones (such as brain, head and neck) for IGRT. The workflow for sites involving bony matches is even simpler because the bony match could eliminate the need for obtaining the orthogonal scouts after proper validation. MRCAT has the potential to be extended to other body sites. The algorithm can be easily applied to gyn and rectum malignancies. Gyn external beam planning often involves para-aortic nodes. In this scenario, the algorithm and ExamCard will need to be modified to include larger FOV to include superior-inferior volume of up to L1 for accurate bone classification. Rectum patients with nodal involvement are usually contoured up to L5/S1, so the FOV will not be an issue but the presence of air in the rectum may impact dosimetry because the MRCAT algorithm does not classify air inside the outer body geometry. However, it is expected that this will be an easy algorithm fix. Finally, the biggest limitation of a MR-only workflow is the inability of MRCAT to generate synthetic CT in the presence of hip prostheses, extensive pelvic disease or large body size. This will continue to be the clinical reality until further improvements within MRCAT (or other syn-CT algorithms) and/or MR scanners can be made to take into account hip implants and gradient nonlinearity effects. Future improvements should investigate metal artifact reduction in conjunction with MRCAT syn-CT generation.

## Conclusions

In this study, we have successfully implemented clinical workflows to perform MR-only simulation, planning and treatment localization. Our clinical experience indicates that MR-only planning is feasible in a clinical setting. Future work will be focused on implementing a more robust, motion-insensitive fiducial identification sequence as well as further minimizing MR simulation time by implementing robust 3D isotropic acquisitions for contouring. Future work will also include developing an MR-based prostate atlas for auto-contouring.

## Additional file

Additional file 1: Figure S1. Effect of motion on Goldseed sequence acquisition. **Figure S2.** Post MR scan QA questionnaire as a document in ARIA. **Figure S3.** Goldseed and MRCAT source MR registered to the small FOV CT to facilitate differentiating gold seed fiducials from brachytherapy seeds. **Figure S4.** Example of a Physics verification image layout in MIM. **Figure S5.** An axial view of MRCAT syn-CT and MRCAT source MR used to identify the 3 external BBs and thereby define the treatment isocenter. (DOCX 8321 kb)

## Abbreviations

2-D: 2-dimensional
3 T: 3 Tesla
3-D: 3-dimensional
AP: Anterior-posterior
bFFE: balanced fast field echo
BW: Bandwidth
CBCT: Cone beam computed tomography
CT: Computed tomography
CTV: Clinical target volume
DRR: Digitally reconstructed radiograph
ELPS: External laser positioning system
FA: Flip angle
FFE: Fast field echo
FH: Foot-head
FOR: Frame of reference
FOV: Field of view
HU: Hounsfield unit
Hz: Hertz
KV: Kilo voltage
LR: Left-right
mm: Millimeter
MRCAT: MR for Calculating attenuation
MRI: Magnetic resonance imaging
NSA: Number of signal averages
PIQT: Periodic image quality test
QA: Quality assurance
ROI: Region of interest
SBRT: Stereotactic body radiation therapy
Syn-CT: Synthetic CT
T2w: T2 weighted
TE: Echo time
TPS: Treatment planning system
TR: Relaxation time
TSE: Turbo spin echo
VMAT: Volumetric modulated arc therapy

## Footnotes

1 Philips Healthcare
