# Supplementary material for: Clinical workflow for MR-only simulation and planning in prostate
Source: Radiat Oncol. 2017 Jul 17;12:119. doi: 10.1186/s13014-017-0854-4 (PMC5513123; doi:10.1186/s13014-017-0854-4)
Supplement: Additional file 1: Figure S1. — Effect of motion on Goldseed sequence acquisition. Figure S2. Post MR scan QA questionnaire as a document in ARIA. Figure S3. Goldseed and MRCAT source MR registered to the small FOV CT to facilitate differentiating gold seed fiducials from brachytherapy seeds. Figure S4. Example of a Physics verification image layout in MIM. Figure S5. An axial view of MRCAT syn-CT and MRCAT source MR used to identify the 3 external BBs and thereby define the treatment isocenter. (DOCX 8321 kb) [file 13014_2017_854_MOESM1_ESM.docx]

Additional file 1

Figure S1: Effect of motion on Goldseed sequence acquisition

Figure S2: Post MR scan QA questionnaire as a document in ARIA.

Figure S3: Goldseed and MRCAT source MR registered to the small FOV CT to facilitate differentiating gold seed fiducials from brachytherapy seeds

Figure S4: Example of a Physics verification image layout in MIM


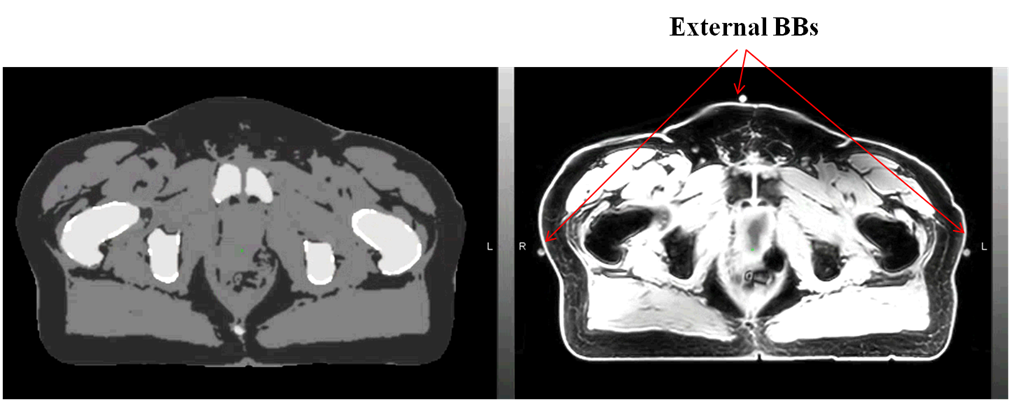


Figure S5: An axial view of MRCAT syn-CT and MRCAT source MR used to identify the 3 external BBs and thereby define the treatment isocenter.
